# Synergistic mitochondrial impairment by endogenously elevated cyanide and hydrogen sulfide in Down syndrome; commentary on: Cyanide overproduction impairs cellular bioenergetics in Down syndrome

**DOI:** 10.1016/j.neurot.2025.e00757

**Published:** 2025-09-25

**Authors:** Andrew A. Pieper, Bindu D. Paul

**Affiliations:** a Department of Psychiatry, Case Western Reserve University, Cleveland, OH, USA; b Geriatric Psychiatry, GRECC, Louis Stokes VA Medical Center, Cleveland, OH, USA; c Institute for Transformative Molecular Medicine, School of Medicine, Case Western Reserve University, Cleveland, OH, USA; d Department of Pathology, Case Western Reserve University, Cleveland, OH, USA; e Brain Health Medicines Center, Harrington Discovery Institute, University Hospitals Cleveland Medical Center, Cleveland, OH, USA; f Department of Neurosciences, Case Western Reserve University, Cleveland, OH, USA; g Department of Physiology, Pharmacology & Therapeutics, Johns Hopkins University School of Medicine, Baltimore, MD, USA; h Department of Psychiatry and Behavioral Sciences, Johns Hopkins University School of Medicine, Baltimore, MD, USA; i The Solomon H. Snyder Department of Neuroscience, Johns Hopkins University School of Medicine, Baltimore, MD, USA; j Lieber Institute for Brain Development, Baltimore, MD, USA

**Keywords:** Down syndrome, Mitochondria, Cyanide, Hydrogen sulfide, Lysosome

Down syndrome (DS), caused by trisomy of chromosome 21, is the most common survivable autosomal aneuploidy [1,2]. Most people with DS (∼95 ​%) carry a full extra copy of chromosome 21 (Hsa21) in all cells, while ∼3–4 ​% have partial triplication of Hsa21 in a subset of cells (mosaicism) [3]. DS affects multiple aspects of physiology, especially the cardiovascular, musculoskeletal, and neurological systems [[4], [5], [6]], and is associated with biochemical abnormalities, dysmorphic features, intellectual disability, and accelerated aging. Individuals with DS also have markedly increased risk of Alzheimer's disease (AD), and DS is recognized as a genetic cause for AD [7,8].

Although the molecular mechanisms underlying DS pathogenesis are not fully understood, abnormalities in mitochondrial gene expression, morphology, and function are well-established hallmarks of the disorder [[9], [10], [11]]. Recent work also implicates dysregulated gasotransmitter signaling as a complementary contributor to mitochondrial compromise in DS [11,12]. Gasotransmitters are gaseous molecules that mediate signaling processes both in the brain and periphery, and include hydrogen sulfide (H_2_S), nitric oxide (NO), carbon monoxide (CO), and hydrogen cyanide (HCN) [[13], [14], [15], [16]]. Their signaling is complex, with both synergistic and antagonistic interactions, and a major component of this process involves posttranslational modification of proteins. This was discovered in 1980 for NO with subsequent characterization and designation as S-nitrosylation [[17], [18], [19], [20], [21], [22], [23]]. Similarly, modification by sulfides was reported in 1998 for H_2_S [24] with extensive characterization of S-sulfhydration/persulfidation in 2009 and beyond [[25], [26], [27], [28]]. S-cyanylation, mediated by HCN, was reported in 2019 in plants and in 2025 in mammalian cells [29,30]. Additionally, CO was reported in 2002 to potentially modify histidine residues of proteins [31]. In this issue of Neurotherapeutics, Petrosino et al. in the Szabo laboratory report that synergistic interaction between H_2_S and HCN disrupts mitochondrial function in DS, providing new mechanistic insight and potential therapeutic directions for this condition.

Once considered purely an environmental toxin, H_2_S is now known to be endogenously generated in mammals and to mediate a wide spectrum of physiological processes, including stress responses, mitochondrial homeostasis, neuronal signaling, cell proliferation, and cardiovascular function [32,33]. H_2_S is produced in mammals mainly by three enzymes, cystathionine γ-lyase (CSE), cystathionine β-synthase (CBS), and 3-mercaptopyruvate sulfur transferase (3-MST), although other non-enzymatic sources of endogenous H_2_S also exist [14]. The physiological effects of H_2_S are dose-dependent, following a biphasic, bell-shaped response in which low concentrations are beneficial and high concentrations are toxic.

The toxic effects of high H_2_S levels, whether from endogenous overproduction or excessive environmental exposure, stem from inhibition of mitochondrial complex IV (cytochrome C oxidase), which impairs ATP production [34,35]. Similarly, ingested cyanide is also toxic to mitochondria by irreversibly binding complex IV [[36], [37], [38]]. The word “cyanide” commonly evokes historical and forensic associations with lethal poisoning [39], and this chemical has been implicated in deaths and chemical warfare for ages. Notable historic events include the 1978 Jonestown massacre and the 1982 Tylenol poisonings [40].

Cyanide was first identified in 1782 by Carl Wilhelm Scheele [41], who is also credited with isolating H_2_S as a distinct chemical species in 1775 [42]. Mammalian tissues synthesize cyanide from glycine, primarily via lysosomal peroxidase activity [30,43]. As a weak acid (pKa 9.2), cyanide at physiological pH exists predominantly (∼95 ​%) as undissociated gaseous HCN, with the remainder as the cyanide anion (CN-). For simplicity, this mixture is referred to as “cyanide.” Low concentrations of cyanide stimulate mitochondrial bioenergetics, whereas higher concentrations inhibit it [30,44,45]. Because of its small molecular size and high solubility, cyanide readily crosses mucous membranes and is rapidly absorbed. Cellular cyanide levels are tightly controlled by the mitochondrial enzyme thiosulfate sulfurtransferase (TST or rhodanese), a key component of the mitochondrial sulfide oxidizing unit and the principal cyanide-detoxifying enzyme [30,33].

The signaling role of cyanide is mediated by post-translational S-cyanylation of cysteine residues in cellular proteins in a manner analogous to H_2_S-mediated persulfidation/sulfhydration and NO-mediated S-nitrosylation [13,29,30,46]. S-cyanylation comprises the addition of CN groups to cysteine residues to form –SCN groups and was first reported in plants [29]. Notably, S-cyanylation does not proceed directly on thiol groups, which must be oxidized first. S-cyanylation affects diverse proteins with broad physiological roles, including metabolism, cytoskeletal architecture, mitochondrial function, protein translation, and transport. Thus, cyanide and H_2_S biology share numerous similarities (Table 1), including dysregulation in DS.

**Table 1:** Common features of HCN and H_2_S.

Property/Action	H_2_S	HCN
Present in the environment of ancient earth	Yes	Yes
Bacterial, mammalian and plant production	Yes	Yes
Amino acid precursor	Yes (cysteine)	Yes (glycine)
Inhibits cytochrome *c* oxidase (complex IV) of mitochondria at high concentrations	Yes	Yes
Stimulates mitochondrial bioenergetics at high concentrations	Yes	Yes
Reacts with metal centers	Yes	Yes
Reacts with hemoglobin	Yes, forms sulfhemoglobin	Yes, forms cyanmethemoglobin
Bell-shaped dose response	Yes	Yes
Neuromodulatory roles: Action on *N*-methyl d-aspartate receptors	Yes	Yes
Mediates posttranslational modification	Yes, on cysteine residues (S-sulfhydration/persulfidation)	Yes, on cysteine residues (S-cyanylation)
Increased in down syndrome	Yes	Yes
Common detoxification enzyme	Yes (TST is one component of the sulfide oxidizing unit in the mitochondria, along with sulfide quinone oxidoreductase (SQR)	Yes (mainly TST; 3-MST and SQR may also metabolize cyanide)
Elimination route	Through urine and exhaled air	Through urine and exhaled air

The Szabo group focused on mitochondrial function, which is impaired in DS and leads to pseudohypoxia and reduced ATP production [47]. The evidence implicating the concerted action of the two gasotransmitters is compelling, with both H_2_S and cyanide levels markedly elevated in DS [30,[48], [49], [50]]. H_2_S is increased in DS due to trisomy of Chr 21, where CBS resides, and to upregulation of 3-MST, the basis for which remains unclear [51,52]. Decreased TST, a component of H_2_S and cyanide clearance, in DS also elevates the accumulation of both gasotransmitters [53]. Petrosino et al. show that TST levels and activity are decreased in DS fibroblasts, accompanied by higher basal and glycine-stimulated cyanide production. They also report increased cyanide levels in the liver, skeletal muscle and brain homogenates as well as blood from the transgenic Sprague-Dawley Dup (Rno20)Yah rat model of DS [50]. Furthermore, elevated cyanide levels were also increased in human DS fibroblasts [45]. Using pharmacological approaches, the team inhibited cyanide production with two different serine hydroxymethyltransferase (SHMT) inhibitors that blocked conversion of serine to glycine, iSHMT and lometrexol hydrate, and observed that this intervention improved mitochondrial function and proliferation in DS cells.

Because HCN production depends on the acidic lysosomal environment and peroxidase activity, lysosomal deacidification with hydroxychloroquine or inhibition of peroxidases with phloroglucinol produced similar benefits, confirming the detrimental effects of excess cyanide on DS cells. Thus, mitochondria in DS appear to be subjected to a double hit from excessive exposure to both H_2_S and cyanide (Fig. 1).

**Fig. 1:**
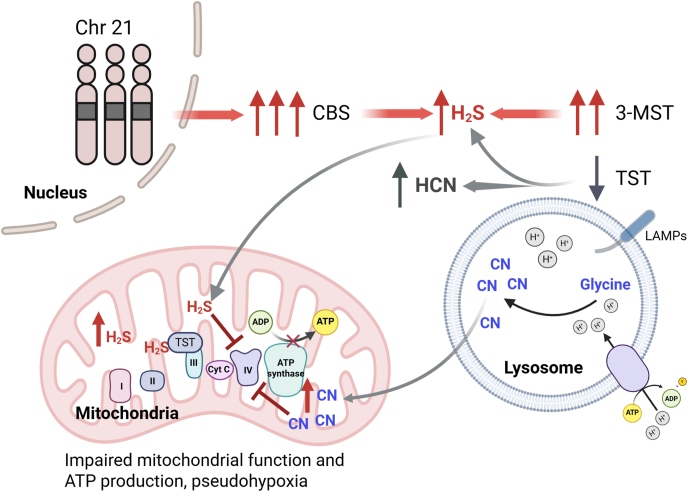
Synergistic action of H_2_S and cyanide on mitochondrial bioenergetics. In Down syndrome, trisomy of chromosome 21 leads to overexpression of cystathionine β synthase (CBS). Overexpression of 3-mercaptopyruvate sulfur transferase (3-MST) also occurs by unknown mechanisms, leading to elevated H_2_S levels that inhibit complex IV of the electron transport chain, thereby decreasing ATP production. DS is also associated with increased cyanide production, which is dependent on lysosomal peroxidases and acidic pH. Cyanide synergizes with H_2_S to further inhibit mitochondrial complex IV and impair mitochondrial bioenergetics.

This study by Petrosino et al. is the first to investigate the concerted actions of these two gasotransmitters in DS. Building on prior reports from the Szabo laboratory showing that inhibiting or silencing CBS is beneficial [49,50,52,53], the present work examined the effects of modulating both H_2_S and cyanide. Additive effects of GYY4137 (a slow release H_2_S donor) and cyanide were observed across dose combinations, producing near complete inhibition of complex IV activity. The findings open new avenues for conceptual and therapeutic investigations. How do endogenous cyanide and S-cyanylation change in different disease states? Are there mammalian enzymes that specifically mediate S-cyanylation? Is there reciprocity between protein persulfidation/sulfhydration mediated by H_2_S and protein S-cyanylation?

Consideration of how cyanide interacts with other gasotransmitters, such as NO or CO, is also important. Cyanide has multiple interactions with NO and H_2_S [36]. For example, NO modulates cyanide's inhibition of mitochondrial complex IV, and low concentrations of cyanide upregulate nitric oxide synthase expression [54,55]. Cyanide also influences H_2_S signaling through several pathways. Thiocyanate can be formed nonenzymatically by reactions of cyanide with elemental sulfur, polysulfides, or sulfide, or enzymatically when 3-MST converts cyanide and thiosulfate (an H_2_S metabolite) to thiocyanate. Thus, 3-MST functions as both an H_2_S-producing enzyme and as a cyanide detoxifying enzyme, meaning that inactivation or decreased expression of 3-MST would simultaneously impair cyanide metabolism and H_2_S production [36]. In DS, elevated cyanide levels may partly reflect decreased TST expression despite increased 3-MST, although other factors such as altered glycine metabolism, peroxidase activity, or lysosomal dysfunction could also contribute. Furthermore, persulfides can react directly with nucleophiles like cyanide to form thiocyanate, potentially modifying persulfide signaling. These interactions warrant further investigation. Finally, therapeutics that harness the beneficial effects of low concentrations of cyanide and H_2_S may offer new approaches to treating neurodegenerative and neurodevelopmental diseases.

## Author contributions

A.A.P. and B.D.P. conceived and wrote the manuscript. B.D.P. created figures. Both authors have read and agreed to the published version of the manuscript.

## Declaration of competing interest

The authors declare that they have no known competing financial interests or personal relationships that could have appeared to influence the work reported in this paper.

## Abbreviations

AD: Alzheimer's disease
CBS: cystathionine β-synthase
CO: carbon monoxide
CSE: cystathionine γ-lyase
DS: Down syndrome
H_2_S: hydrogen sulfide
HCN: hydrogen cyanide
Hsa21: human chromosome 21
3-MST: 3-mercaptopyruvate sulfurtransferase
NO: nitric oxide
SHMT: serine hydroxymethyltransferase
iSHMT: inhibitor of serine hydroxymethyltransferase
SQR: sulfide quinone oxidoreductase
TST: thiosulfate sulfurtransferase
